# Dynamics of Frenkel Excitons in Pentacene

**DOI:** 10.3390/ma11112219

**Published:** 2018-11-08

**Authors:** Sonja Gombar, Petar Mali, Milan Pantić, Milica Pavkov-Hrvojević, Slobodan Radošević

**Affiliations:** Department of Physics, Faculty of Sciences, University of Novi Sad, Trg Dositeja Obradovića 4, 21000 Novi Sad, Serbia; df.sonja.gombar2@student.pmf.uns.ac.rs (S.G.); mpantic@df.uns.ac.rs (M.P.); milica@df.uns.ac.rs (M.P.-H.); slobodan@df.uns.ac.rs (S.R.)

**Keywords:** Frenkel excitons, Heisenberg model, effective field theory, exciton-exciton interactions

## Abstract

The dispersion relation for noninteracting excitons and the influence of perturbative corrections are examined in the case of pentacene structure. The values of exchange integrals are determined by nonlinear fits to the experimental dispersion data, obtained by the inelastic electron scattering reported in recent experiments. We obtain theoretical dispersion curves along four different directions in the Brillouin zone which possess the same periodicity as the experimental data. We also show that perturbative corrections are negligible since the exciton gap in the dispersion relation is huge in comparison to the exchange integrals.

## 1. Introduction

In the last few decades organic molecular solids have been a matter of intense theoretical and experimental study, due to their potential applications in novel organic devices [1,2]. The recent advances in experimental methods have provided detailed insight into their microscopic properties [3]. Among the energetically lowest excitations in such systems are Frenkel excitons; electron–hole pairs of small radius [4]. The general theory of Frenkel excitons in molecular crystals is described in detail in Reference [5], and some references on applications and progress are provided in References [6,7,8,9].

The method of inelastic electron scattering was used for direct measurement of the exciton band structure within the reciprocal $a^{*}b^{*}$ plane of pentacene at room temperature (T=300 K) in Reference [3]. Results of measurements along four different directions in the Brillouin zone were presented and, on that basis, the authors of Reference [3] argued that the model of noninteracting Frenkel excitons is inapplicable for description of pentacene (see also Reference [10] for measurements at T=20 K and Reference [11] for similar experiments on picene). They also suggested that charge-transfer (CT) excitons must be included in the model Hamiltonian of pentacene in order to achieve better agreement with experiments. Following these experiments, a significant theoretical work was conducted in order to obtain the properties of pentacene from first principles, i.e., starting from many-body electron–hole Hamiltonians [12,13,14,15,16].

The present paper deals with the problem of obtaining exciton dispersion in pentacene by relying on a correspondence between the Paulion Hamiltonian and anisotropic XXZ Heisenberg ferromagnets. Unlike previous theoretical work, based on many-body Hamiltonians containing electron and hole creation and annihilation operators, we present calculations based on an effective Hamiltonian [17,18,19]. In other words, we start from Frenkel excitons as low lying degrees of freedom and obtain an effective form of their interactions, which are considered to the one-loop order.

Whereas our results confirm that exciton dispersion in pentacene cannot be satisfactorily described within the Frenkel model alone, they also suggest that the influence of other excitations may not be as large as originally proposed.

The paper is organized as follows. The model Hamiltonian and pentacene structure are introduced in Section 2, while exciton dispersion in noninteracting model is obtained in Section 3. Finally, we discuss the effects of exciton–exciton interactions within the two-level model in Section 4 and Section 5.

## 2. Model Hamiltonian and Pentacene Structure

The basic Hamiltonian that governs the dynamics of excitons in a two-level system (only one electronically excited molecular state is considered) is given by:

(1) $$\begin{array}{ccc} H & = & H_{0}+\Delta \sum_{n}P_{n}^{+}P_{n}-\frac{X}{2}\sum_{n,\lambda }P_{n}^{+}P_{n+\lambda }\\ & - & \frac{Y}{2}\sum_{n,\lambda }P_{n}^{+}P_{n}P_{n+\lambda }^{+}P_{n+\lambda }, \end{array}$$

where $P_{n}^{+}$ and $P_{n}$ are standard Pauli operators on site n, and *X* and *Y* are parameters describing hopping and interactions of excitons, respectively [5,20]. Using the exact one-to-one correspondence between Pauli and spin operators in the case of S=1/2 [21], we obtain the anisotropic (XXZ) Heisenberg Hamiltonian in an external field:

(2) $$H=-\frac{I^{x}}{2}\sum_{n,\lambda }S_{n}^{-}S_{n+\lambda }^{+}-\frac{I^{z}}{2}\sum_{n,\lambda }S_{n}^{z}S_{n+\lambda }^{z}-\mu H\sum_{n}S_{n}^{z},$$

where {λ} denotes vectors connecting neighboring sites, $z_{1}$ is the number of nearest neighbors, and:

(3) $$\begin{array}{c} I^{z}=Y,\;I^{x}=X,\;\mu H=\Delta -\frac{I^{z}z_{1}}{2}. \end{array}$$

Equivalently, the inverse relations are:

(4) $$\begin{array}{ccc} \Delta & = & \frac{I^{z}z_{1}}{2}+\mu H,\;Y=I^{z},\;X=I^{x},\;\\ H_{0} & = & -\frac{I^{z}Nz_{1}}{8}-\frac{N\mu H}{2}. \end{array}$$

Due to the isomorphism of the spin and Paulion Hilbert spaces on every lattice site and the relations in Equations (3) and (4), the original problem of exciton dynamics governed by Equation (1) can be completely mapped onto the equivalent effective spin model in Equation (2). It should be noted that this correspondence is purely formal—it will allow us to investigate the exciton system with the help of a vast number of existing theoretical tools developed for spin systems [21,22,23,24,25,26,27,28,29]. According to Reference [5], the Pauli Hamiltonian of Equation (1), which is as we have shown here equivalent to the anisotropic Heisenberg Hamiltonian of Equation (2), can be used in the description of pentacene. This fact will enable us to examine the dispersion of noninteracting excitons as well as the influence of their interactions with a leading order (one-loop) approximation. One should also note that, in many practical cases, the set of neighboring sites connected with hopping integrals splits into several subsets, determined by the lattice structure and values of the hopping parameters.

**Figure 1:**
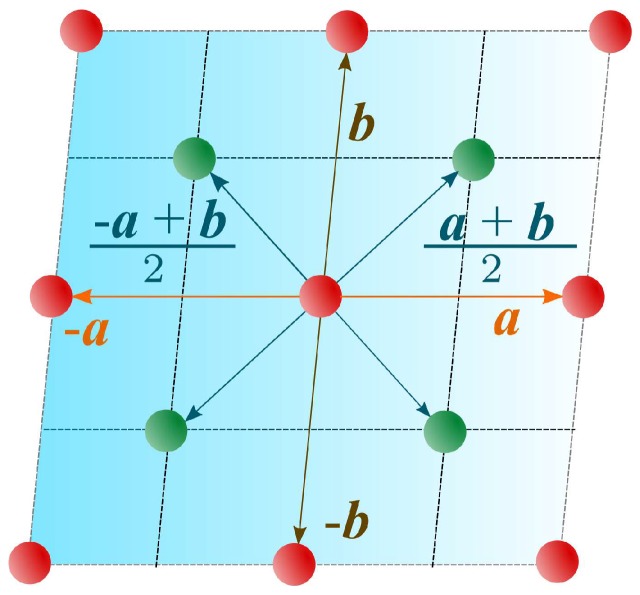
Schematic presentation of the pentacene lattice. A pair of exchange integrals corresponds to each set of the lattice vectors {a,−a}, {b,−b}, and $\left\{\frac{a+b}{2},\frac{-a+b}{2},-\frac{a+b}{2},-\frac{-a+b}{2}\right\}$ (see the text).

We shall analyze now the pentacene structure. A schematic sketch of a pentacene thin film lattice is shown in Figure 1. The lattice parameters within the ab layer of the single crystal of pentacene are $\left|a\right|=6.27\;\overset{˚}{A}$, $\left|b\right|=7.78\;\overset{˚}{A}$, ∢(a,b)=87.8° [30]. The central motive in Figure 1 has three types of neighbors: two neighbors at points $\lambda_{1}=\left\{a,-a\right\}$ coupled trough the exchange integral $I_{1}$, two neighbors at points $\lambda_{2}=\left\{b,-b\right\}$ coupled through the exchange integral $I_{2}$ and four neighbors at points $\lambda_{3}=\left\{\frac{a+b}{2},\frac{-a+b}{2},-\frac{a+b}{2},-\frac{-a+b}{2}\right\}$ coupled via the exchange integral $I_{3}$. As we have already noted, the transition from Pauli to Heisenberg Hamiltonian requires anisotropic exchange integrals. Therefore, each of the mentioned exchange integrals splits into *x* and *z* components: $I_{j}\to \left(I_{j}^{x},I_{j}^{z}\right)$, where j=1,2 or 3. The results from a recent paper [31] showed that the most important hopping paths in the pentacene crystal are in the planes perpendicular to the $c^{*}$ axis. Thus, to a good approximation, the real pentacene crystal can be effectively described by a two dimensional model. Finally, by following common practice [3], we shall present numerical results for the approximate pentacene lattice, defined by the additional constraint a·b=0.

## 3. Dispersion of Noninteracting Excitons

Bearing in mind the remarks on pentacene structure from the previous section, we obtain the Hamiltonian of Equation (2), adapted to the pentacene structure, which in the Bloch approximation is:

(5) $$\begin{array}{ccc} H & = & H_{0}^{\prime }-\frac{1}{2}\sum_{j}I_{j}^{x}\sum_{n,\lambda_{j}}B_{n}^{†}B_{n+\lambda_{j}}+\frac{1}{2}\sum_{j}I_{j}^{z}\sum_{n,\lambda_{j}}B_{n}^{†}B_{n}\\ & + & \mu H\sum_{n}B_{n}^{†}B_{n}\\ & = & H_{0}^{\prime }-\frac{I_{1}^{x}}{2}\sum_{n,\lambda_{1}}B_{n}^{†}B_{n+\lambda_{1}}+\frac{I_{1}^{z}}{2}\sum_{n,\lambda_{1}}B_{n}^{†}B_{n}\\ & - & \frac{I_{2}^{x}}{2}\sum_{n,\lambda_{2}}B_{n}^{†}B_{n+\lambda_{2}}+\frac{I_{2}^{z}}{2}\sum_{n,\lambda_{2}}B_{n}^{†}B_{n}\\ & - & \frac{I_{3}^{x}}{2}\sum_{n,\lambda_{3}}B_{n}^{†}B_{n+\lambda_{3}}+\frac{I_{3}^{z}}{2}\sum_{n,\lambda_{3}}B_{n}^{†}B_{n}\\ & + & \mu H\sum_{n}B_{n}^{†}B_{n}, \end{array}$$

where $B_{n}^{†}\;\left(B_{n}\right)$ are Boson creation (annihilation) operators. The same Hamiltonian in k space has the form:

(6) $$\begin{array}{ccc} \tilde{H} & = & {\tilde{H}}_{0}^{\prime }-\frac{I_{1}^{x}}{2}\sum_{k}B_{k}^{†}B_{k}z_{1}\gamma_{1}\left(k\right)+\frac{I_{1}^{z}z_{1}}{2}\sum_{k}B_{k}^{†}B_{k}\\ & - & \frac{I_{2}^{x}}{2}\sum_{k}B_{k}^{†}B_{k}z_{2}\gamma_{2}\left(k\right)+\frac{I_{2}^{z}z_{2}}{2}\sum_{k}B_{k}^{†}B_{k}\\ & - & \frac{I_{3}^{x}}{2}\sum_{k}B_{k}^{†}B_{k}z_{3}\gamma_{3}\left(k\right)+\frac{I_{3}^{z}z_{3}}{2}\sum_{k}B_{k}^{†}B_{k}\\ & + & \mu H\sum_{k}B_{k}^{†}B_{k}, \end{array}$$

where $z_{1}=z_{2}=2$, $z_{3}=4$ and the corresponding geometric factors are defined by:

(7) $$\gamma_{1}\left(k\right)=\frac{1}{2}\sum_{\lambda_{1}}e^{ik\cdot \lambda_{1}}=\frac{1}{2}\left(e^{ik\cdot a}+e^{-ik\cdot a}\right)=\cos\left(k\cdot a\right),$$

(8) $$\gamma_{2}\left(k\right)=\frac{1}{2}\sum_{\lambda_{2}}e^{ik\cdot \lambda_{2}}=\frac{1}{2}\left(e^{ik\cdot b}+e^{-ik\cdot b}\right)=\cos\left(k\cdot b\right),$$

(9) $$\begin{array}{ccc} \gamma_{3}\left(k\right) & = & \frac{1}{4}\sum_{\lambda_{3}}e^{ik\cdot \lambda_{3}}\\ & = & \frac{1}{4}\left(e^{ik\cdot \frac{a+b}{2}}+e^{-ik\cdot \frac{a+b}{2}}+e^{ik\cdot \frac{a-b}{2}}+e^{-ik\cdot \frac{a-b}{2}}\right)\\ & = & \frac{1}{2}\cos\left[\frac{k\cdot (a+b)}{2}\right]+\frac{1}{2}\cos\left[\frac{k\cdot (a-b)}{2}\right]\\ & = & \cos\left(\frac{k\cdot a}{2}\right)\cos\left(\frac{k\cdot b}{2}\right). \end{array}$$

From:

(10) $$\tilde{H}={\tilde{H}}_{0}^{\prime }+\sum_{k}E\left(k\right)B_{k}^{†}B_{k},$$

we obtain the dispersion relation:

$$\begin{array}{ccc} E(k) & = & I_{1}^{x}\left[\frac{I_{1}^{z}}{I_{1}^{x}}-\cos\left(k\cdot a\right)\right]+I_{2}^{x}\left[\frac{I_{2}^{z}}{I_{2}^{x}}-\cos\left(k\cdot b\right)\right]\\ & + & 2I_{3}^{x}\left[\frac{I_{3}^{z}}{I_{3}^{x}}-\cos\left(\frac{k\cdot a}{2}\right)\cos\left(\frac{k\cdot b}{2}\right)\right]+\mu H. \end{array}$$

That is:

(11) $$\begin{array}{ccc} E(k) & = & \Delta -I_{1}^{x}\cos\left(k\cdot a\right)-I_{2}^{x}\cos\left(k\cdot b\right)\\ & - & 2I_{3}^{x}\cos\left(\frac{k\cdot a}{2}\right)\cos\left(\frac{k\cdot b}{2}\right), \end{array}$$

where we have defined the exciton gap:

(12) $$\Delta =I_{1}^{z}+I_{2}^{z}+2I_{3}^{z}+\mu H.$$

**Figure 2:**
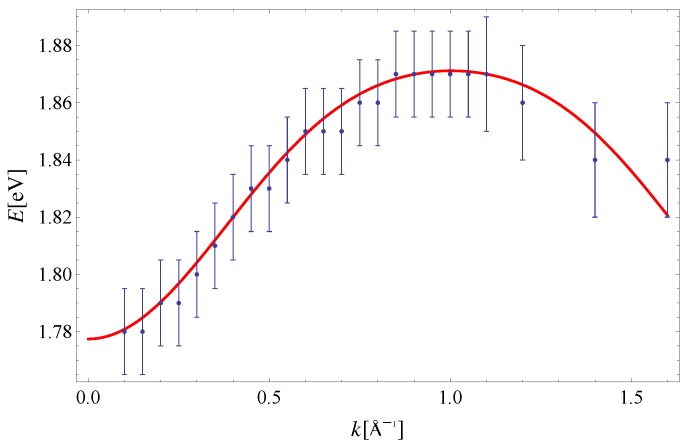
Exciton dispersion along the (100) direction. Experimental data are taken from Reference [3]. The theoretical curve is obtained for: Δ=1.83eV [33], $I_{1}^{x}=5.7\;\mathrm{meV}$ and $I_{3}^{x}=23.4\;\mathrm{meV}$.

**Figure 3:**
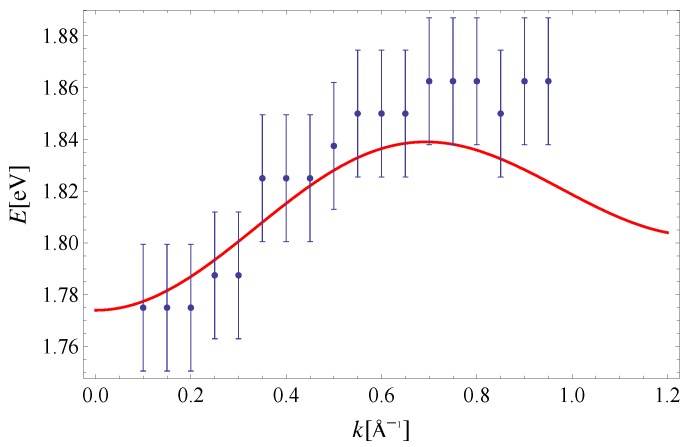
Exciton dispersion along the (210) direction. Experimental data are taken from Reference [3]. The theoretical curve is obtained for: $I_{2}^{x}=3.4\;\mathrm{meV}$ and the remaining parameters are the same as in Figure 2.

**Figure 4:**
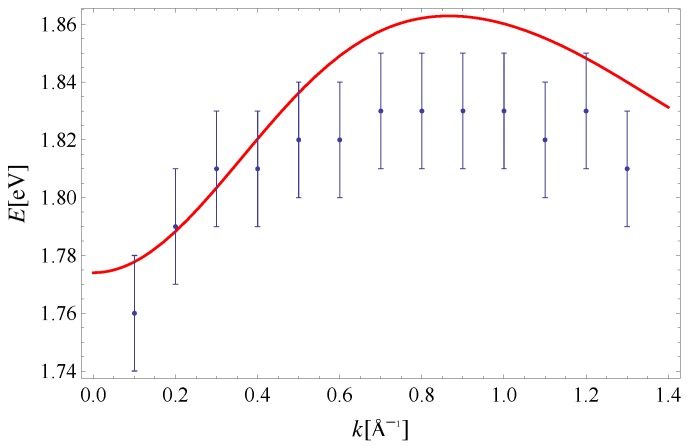
Exciton dispersion along the (120) direction. Experimental data are taken from Reference [3]. The parameters used for the theoretical fit are as in Figure 2 and Figure 3.

**Figure 5:**
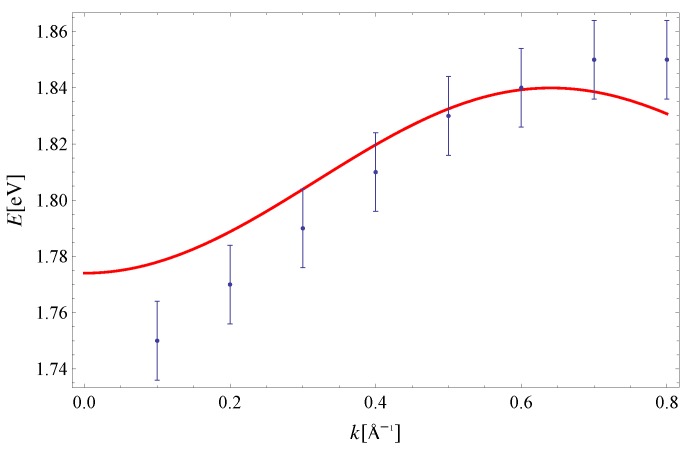
Exciton dispersion along the (110) direction. Experimental data are taken from Reference [3]. The parameters used for the theoretical fit are as in Figure 2 and Figure 3.

**Figure 6:**
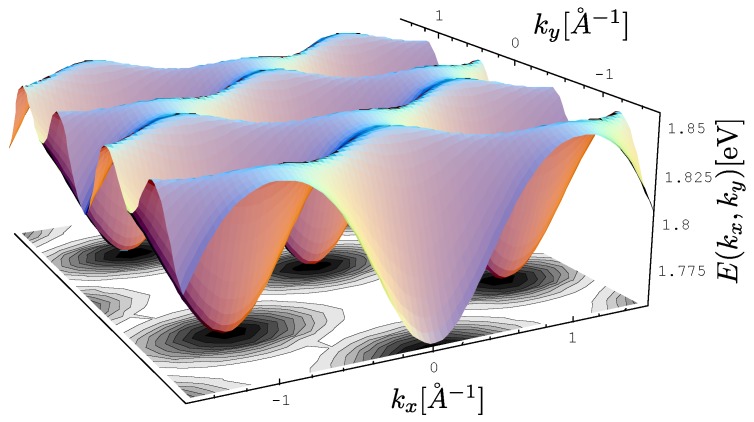
Exciton dispersion in three dimensions. The parameters used for the theoretical fit are as in Figure 2 and Figure 3.

The exciton dispersion law of Equation (11) is plotted along (100) in Figure 2. Note that the orthogonality condition a·b=0 allows us to determine $I_{1}^{x}=5.7\;\mathrm{meV}$ and $I_{3}^{x}=23.4\;\mathrm{meV}$ by fitting Equation (11) to experimental data along this direction only (see Reference [32] for a discussion on the determination of exchange integrals from dispersion relations in a similar context). The value Δ=1.83eV is taken from [33]. The last parameter $I_{2}^{x}=3.4\;\mathrm{meV}$ is extracted from the experimental data on exciton dispersion along the (210) direction (see Figure 3). By using this set of parameters, we have plotted the exciton dispersion along the (110) and (120) directions and compared them to the experimental data from Reference [3]. Since we have used a single set of model parameters, the plotted dispersion law displays the unique limit $\Delta -I_{1}^{x}-I_{2}^{x}-2I_{3}^{x}=1.7741\;\mathrm{eV}$ as |k|→0 for all four directions in the Brillouin zone. This is clearly seen from Figure 2, Figure 3, Figure 4 and Figure 5. Finally, a 3D plot of the exciton dispersion $E(k_{x},k_{y})$ is given in Figure 6.

The disagreement between the dispersion of excitons predicted by the noninteracting Frenkel model and the experimental data, which is evident from Figure 2, Figure 3, Figure 4 and Figure 5, should be attributed to the existence of other excitations (CT excitons) in the system, according to Reference [3]. Specifically, the difference between the theoretical curve and experiment was the most prominent along the (120) direction in Reference [3], since the calculated and measured dispersions do not share the same periodicity. Even though the agreement between theory and experiment is the best for the (100) direction, the theoretical curves presented here possess the same periodicity as the experimental data within the Brillouin zone, for all four directions. Thus, the influence of CT excitons may not be as large as originally suggested in Reference [3].

The exciton dispersion in Reference [3] is given by:

(13) $$\begin{array}{ccc} E(k) & = & E_{0}+t_{a}\cos\left(k\cdot a\right)+t_{b}\cos\left(k\cdot b\right)\\ & + & 2t_{ab}\cos\left(\frac{k\cdot a}{2}\right)\cos\left(\frac{k\cdot b}{2}\right). \end{array}$$

Since the effective mass of excitons in pentacene is large [34], the hopping parameters in Equation (1) are positive and small, such that the corresponding Heisenberg Hamiltonian of Equation (2) describes a ferromagnet. By comparing Equations (11) and (13) we find:

(14) $$\begin{array}{c} \;t_{a}=-I_{1}^{x}<0,\;t_{b}=-I_{2}^{x}<0,\;t_{ab}=-I_{3}^{x}<0. \end{array}$$

Therefore, without fitting the dispersion to experimental data, we conclude that $t_{a}$, $t_{b}$, and $t_{ab}$ must be negative, which is in accordance with Reference [3].

## 4. Perturbative Corrections

In the previous section we saw that the model of noninteracting excitons gives a dispersion law which lies within error bars for almost the entire Brillouin zone. The question, which naturally follows this observation is: could the agreement between theory and experiment be improved by including the effects of exciton–exciton interactions? By answering this question we could provide additional support for the hypothesis proposed for the first time in Reference [3], according to which additional excitations (CT excitons) need to be taken into account for the correct description of pentacene.

A careful examination of traditional methods for studying the effects of interactions in models based on the Pauli/Heisenberg Hamiltonians of Equations (1) and (2) reveals that they possess certain flaws. To avoid them, we employ the perturbation theory developed in References [35,36]. The main advantage of this method is that Boson representations of spin operators are unnecessary. In other words, exciton–exciton interactions, which are partially hidden in the spin Hamiltonian and partially in the corresponding Hilbert space, are explicitly given through interaction pieces of the Lagrangian. Therefore, perturbative corrections may be calculated more systematically. This is extremely important for S=1/2 spin Hamiltonians, since 1/S is not a small parameter that can control perturbative calculations.

As it is well known, the Lagrangian that reproduces the Landau–Lifshitz equation is [18,37,38]:

(15) $$\begin{array}{c} L_{\mathrm{eff}}=\Sigma \frac{\partial_{t}U^{1}U^{2}-\partial_{t}U^{2}U^{1}}{1+U^{3}}-\frac{F^{2}}{2}\partial_{\alpha }U^{i}\partial_{\alpha }U^{i}+\Sigma \mu HU^{3}, \end{array}$$

where two excitation fields are collected into the unit vector $U:={\left[U^{1}\;\;U^{2}\;\;U^{3}\right]}^{T}\equiv {\left[\pi \left(x\right),U^{3}\left(x\right)\right]}^{T}$, Σ=NS/V, and *F* is a constant to be determined later.

The corresponding free part of the Lagrangian is:

(16) $$\begin{array}{c} L_{\mathrm{free}}=\frac{\Sigma }{2}\left[\partial_{t}\pi^{1}\pi^{2}-\partial_{t}\pi^{2}\pi^{1}\right]+\frac{F^{2}}{2}\pi \cdot \partial_{\alpha }\partial_{\alpha }\pi +\Sigma \mu H\pi^{2}, \end{array}$$

and the interaction part up to a quartic approximation (which is sufficient for one-loop calculations) is:

(17) $$L_{\mathrm{int}}=\frac{F^{2}}{8}\pi^{2}\partial_{\alpha }\partial_{\alpha }\pi^{2}-\frac{F^{2}}{8}\pi^{2}\pi \cdot \partial_{\alpha }\partial_{\alpha }\pi .$$

To obtain the Hamiltonian suitable for a perturbative calculation, we apply canonical quantization and incorporate the structure of the lattice presented in Figure 1. Basically, this means that we wish to construct the free Hamiltonian with lattice exciton fields that reproduces the exciton dispersion of Equation (11):

(18) $$H_{0}=-\frac{\upsilon_{0}}{2m}\sum_{x}\psi^{†}D^{2}\psi +\mu H\upsilon_{0}\sum_{x}\psi^{†}\psi ,$$

where $\upsilon_{0}=ab$, ψ and $\psi^{†}$ satisfy canonical commutation relations for Schrodinger fields, and $D^{2}$ and *m* are the discrete Laplacian and a parameter defined in such a way that Equation (18) reproduces the dispersion in Equation (11). It can be readily checked that $D^{2}$ is given by:

(19) $$\begin{array}{ccc} D^{2} & = & \nabla_{\left(3\right)}^{2}+\frac{1}{2}\frac{|\lambda_{1}{|}^{2}}{|\lambda_{3}{|}^{2}}\frac{I_{1}^{x}}{I_{3}^{x}}\nabla_{\left(1\right)}^{2}+\frac{1}{2}\frac{|\lambda_{2}{|}^{2}}{|\lambda_{3}{|}^{2}}\frac{I_{2}^{x}}{I_{3}^{x}}\nabla_{\left(2\right)}^{2}, \end{array}$$

where:

(20) $$\begin{array}{ccc} \nabla_{\left(j\right)}^{2}\phi \left(x\right) & := & \frac{4}{z_{j}{\left|\lambda_{j}\right|}^{2}}\sum_{\lambda_{j}}\left(\phi \left(x+\lambda_{j}\right)-\phi \left(x\right)\right),\\ j & = & 1,2,3 \end{array}$$

are the discrete Laplacians for the three sets of neighbors (see Figure 1) and:

(21) $$\begin{array}{c} m=\frac{1}{I_{3}^{x}{\left|\lambda_{3}\right|}^{2}}\equiv \frac{\Sigma }{2F^{2}}. \end{array}$$

Further, it is useful to introduce the eigenvalues of the discrete Laplacians. They are given by:

(22) $$\begin{array}{c} \nabla_{\left(j\right)}^{2}e^{ik\cdot x}=-{\hat{k}}_{\left(j\right)}^{2}e^{ik\cdot x},\\ {\hat{k}}_{\left(j\right)}^{2}:=\frac{2D}{|\lambda_{j}{|}^{2}}\left(1-\gamma_{j}\left(k\right)\right), \end{array}$$

and the exciton dispersion is obtained from Equation (18) by a Fourier transform, which may be written as:

(23) $$\begin{array}{c} E\left(k\right)=\mu H+\delta_{1}+\delta_{2}+\delta_{3}+\frac{I_{3}^{x}{\left|\lambda_{3}\right|}^{2}}{2}\left[{\hat{k}}_{\left(3\right)}^{2}+\frac{1}{2}\frac{|\lambda_{1}{|}^{2}}{|\lambda_{3}{|}^{2}}\frac{I_{1}^{x}}{I_{3}^{x}}{\hat{k}}_{\left(1\right)}^{2}+\frac{1}{2}\frac{|\lambda_{2}{|}^{2}}{|\lambda_{3}{|}^{2}}\frac{I_{2}^{x}}{I_{3}^{x}}{\hat{k}}_{\left(2\right)}^{2}\right]\equiv \Delta +\frac{{\hat{k}}^{2}}{2m}, \end{array}$$

where $\delta_{j}=I_{j}^{z}-I_{j}^{x}$. Thus, the Hamiltonian of Equation (18) is equivalent to the Bloch Hamiltonian of Equation (10). Note that the discrete Laplacian $D^{2}$, which defines local changes of the excitation fields, depends on the ratios $I_{1}^{x}/I_{3}^{x}$ and $I_{2}^{x}/I_{3}^{z}$. Thus, the full symmetry of the pentacene lattice, which reflects itself through the energies of free excitons, can be implemented within the effective model only by the right choice of exchange integrals. The exciton–exciton interactions, which modify exciton dispersion to the one-loop level, can now be written as (see References [35,36]):

(24) $$H_{\mathrm{int}}=H_{4}^{\left(a\right)}+H_{4}^{\left(b\right)},$$

with:

(25) $$\begin{array}{ccc} H_{4}^{\left(a\right)} & = & \frac{F^{2}}{8}v_{0}\sum_{x}\pi^{2}\left(x\right)\pi \left(x\right)\cdot D^{2}\pi \left(x\right),\\ H_{4}^{\left(b\right)} & = & -\frac{F^{2}}{8}v_{0}\sum_{x}\pi^{2}\left(x\right)D^{2}\pi^{2}\left(x\right), \end{array}$$

and:

(26) $$\begin{array}{ccc} \psi & = & \sqrt{\frac{2}{\Sigma }}\left[\pi^{2}+i\pi^{2}\right]. \end{array}$$

Now we can calculate the one-loop correction to the exciton dispersion. It is determined by the one-loop self energy which, in turn, may be calculated by the diagrammatic rules introduced in References [35,36]. In short, we see from Equation (25) that the excitons interact via derivative couplings, so that internal and external lines on Feynman diagrams carry eigenvalues of the discrete Laplacians [17]. These are denoted by colored propagator lines. The exciton propagator, written in Matsubara formalism, is given by:

(27) $$\begin{array}{ccc} \;D(x-y,\tau_{x}-\tau_{y}) & = & {\langle T\left\{\psi \left(x,\tau_{x}\right)\psi^{†}\left(y,\tau_{y}\right)\right\}\rangle }_{0}\\ & = & \frac{1}{\beta }\sum_{n=-\infty }^{\infty }\int_{q}\frac{e^{iq\cdot \left(x-y\right)-i\omega_{n}\left(\tau_{x}-\tau_{y}\right)}}{E\left(q\right)-i\omega_{n}}, \end{array}$$

where:

(28) $$\begin{array}{c} \int_{q}\equiv \int_{\mathrm{IBZ}}\frac{d^{D}q}{{\left(2\pi \right)}^{D}}, \end{array}$$

and IBZ denotes the first Brillouin zone.

Within the one-loop approximation, there are four types of diagrams. They can be easily evaluated to:
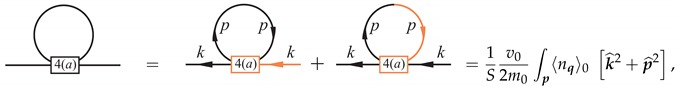
(29)

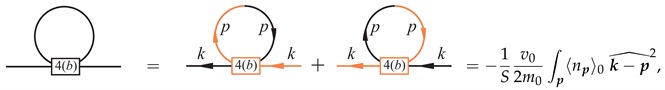
(30)
with ${\hat{k}}^{2}$ defined in Equation (23) and ${\langle n_{p}\rangle }_{0}$ denoting the free exciton Bose distribution. An explicit expression for the exciton self-energy is found by using the relation:

$$\begin{array}{c} \int_{q}{\langle n_{q}\rangle }_{0}\;\hat{p-q}{\;}_{\left(j\right)}^{2}=\int_{q}{\langle n_{q}\rangle }_{0}\left[{\hat{p}}_{\left(j\right)}^{\;2}+{\hat{q}}_{\left(j\right)}^{\;2}-\frac{|\lambda_{j}{|}^{2}}{2D}{\hat{p}}_{\left(j\right)}^{\;2}\;{\hat{q}}_{\left(j\right)}^{\;2}\right], \end{array}$$

and is given by:

(31) $$\begin{array}{c} \Sigma \left(k\right)=\frac{{\hat{k}}_{\left(3\right)}^{2}}{2m}A_{3}\left(T\right)+\frac{{\hat{k}}_{\left(1\right)}^{2}}{2m}A_{1}\left(T\right)+\frac{{\hat{k}}_{\left(2\right)}^{2}}{2m}A_{2}\left(T\right). \end{array}$$

The temperature dependent factors $A_{j}\left(T\right)$ are:

(32) $$\begin{array}{ccc} A_{1}\left(T\right) & = & \frac{a^{2}}{2D}\frac{|\lambda_{1}{|}^{2}}{|\lambda_{3}{|}^{2}}\frac{I_{1}^{x}}{I_{3}^{x}}v_{0}\int_{q}{\langle n_{q}\rangle }_{0}{\hat{q}}_{\left(1\right)}^{\;2},\\ A_{2}\left(T\right) & = & \frac{b^{2}}{2D}\frac{|\lambda_{2}{|}^{2}}{|\lambda_{3}{|}^{2}}\frac{I_{2}^{x}}{I_{3}^{x}}v_{0}\int_{q}{\langle n_{q}\rangle }_{0}{\hat{q}}_{\left(2\right)}^{\;2},\\ A_{3}\left(T\right) & = & \frac{2|\lambda_{3}{|}^{2}}{2D}v_{0}\int_{q}{\langle n_{q}\rangle }_{0}{\hat{q}}_{\left(3\right)}^{\;2}. \end{array}$$

**Figure 7:**
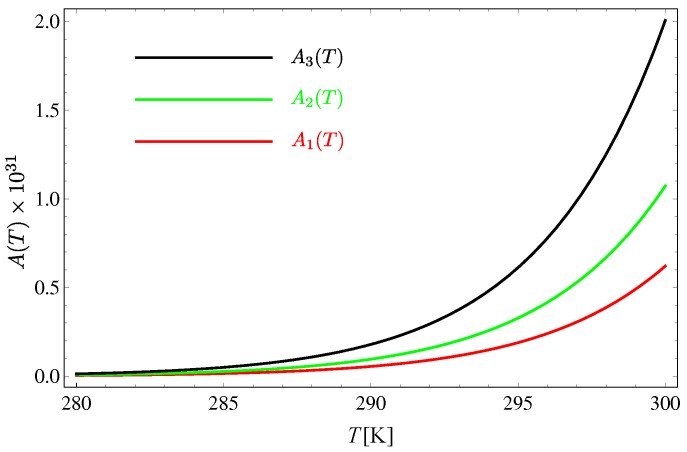
Renormalizing factors $A_{j}\left(T\right)$ defined in Equation (32).

They are dimensionless quantities that capture the effects of exciton–exciton interactions in two-level system by renormalizing the exchange integrals $I_{j}^{x}\to I_{j}^{x}\left(T\right)$. Finally, the renormalized exciton energies are [18]:

(33) $$E_{R}\left(k\right)=E\left(k\right)-\Sigma \left(k\right),$$

and the influence of exciton–exciton interactions at one-loop order can be seen in Figure 7.

**Figure 8:**
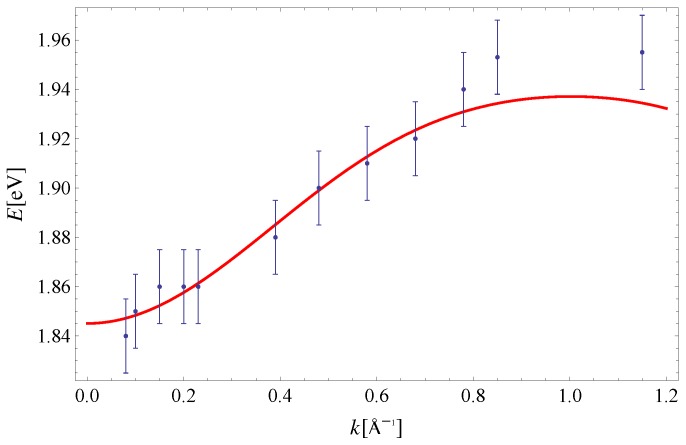
Exciton dispersion along the (100) direction. Experimental data are taken from Reference [10]. The theoretical curve is obtained for: Δ=1.90eV [33], $I_{1}^{x}=5.7\;\mathrm{meV}$, $I_{2}^{x}=3.4\;\mathrm{meV}$ and $I_{3}^{x}=23.4\;\mathrm{meV}$.

**Figure 9:**
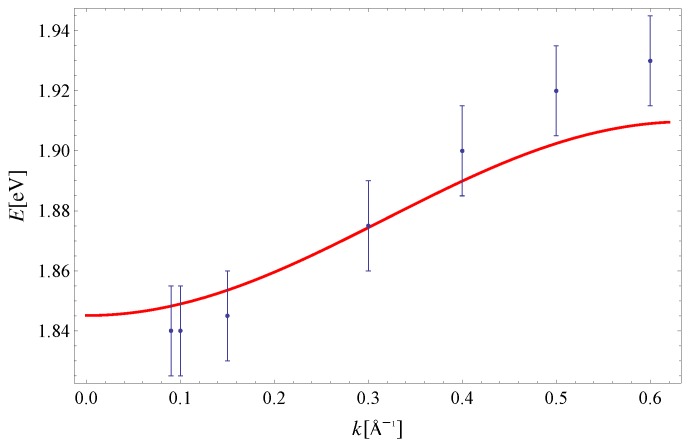
Exciton dispersion along the (110) direction. Experimental data are taken from Reference [10]. The theoretical curve is obtained for: Δ=1.90eV [33], $I_{1}^{x}=5.7\;\mathrm{meV}$, $I_{2}^{x}=3.4\;\mathrm{meV}$ and $I_{3}^{x}=23.4\;\mathrm{meV}$.

As noted in the Introduction, the experimental data on exciton dispersion along the (100) and (110) directions at lower temperatures are available [10]. One can see from Figure 8 and Figure 9 that the effective model of Equation (18), with parameters Δ=1.90eV [33], $I_{1}^{x}=5.7\;\mathrm{meV}$, $I_{2}^{x}=3.4\;\mathrm{meV}$, and $I_{3}^{x}=23.4\;\mathrm{meV}$ gives an exciton dispersion that satisfyingly agrees with the experimental one. The only difference between the two sets of parameters, describing experimental data obtained at 300K and 20K, is the value of the parameter Δ, which originates from the change of magnetic field H in Equation (2).

## 5. Discussion

As seen from Figure 7, the influence of exciton–exciton interactions is negligible at room temperatures. There are two main reasons for this. First, the exciton gap is huge—nearly two orders of magnitude larger than the greatest exchange integral. Second, excitons are derivatively coupled via interactions that are of the type occurring in the nonlinear σ models. Since these interactions include Laplacians, they tend to vanish at low energies. In fact, recent studies [39,40] have shown that scattering amplitudes in a system governed by such interactions disappear as the momenta of particles tend to zero. This interpretation is similar to the one given by Dyson in his analysis of ferromagnetic systems [41,42]. On the other hand, since the scattering amplitudes tend to zero regardless of exciton gap (i.e., the fictitious external magnetic field of the corresponding ferromagnetic system), it contradicts the “hard sphere” picture of exciton dynamics from Reference [20].

It is important to compare results from the present paper to the ones obtained by solving the Bethe–Salpeter (BS) equations for a many-body electron-hole system [13,14]. First, the exciton dispersion obtained here is closer to experimental values. This is clearly seen by comparing Figure 2, Figure 3, Figure 4 and Figure 5 from present paper to the results of Cudazzo et al. (see Figure 3 in Reference [13]). Second, the exciton dispersion obtained with the help of the effective model in the present paper is much more robust against perturbative corrections. This may also be seen from Figures 3 and 4 in Reference [13]; the dispersion obtained using flat HOMO-LUMO bands yields exciton dispersion close to 3.5eV, while those obtained using HOMO-LUMO with full dispersion are between 1.77eV and 1.8eV along the (100) direction.

To conclude, we have analyzed the exciton dispersion in pentacene relying on the correspondence between the Pauli (Equation (1)) and Heisenberg (Equation (2)) Hamiltonians. By fitting exchange integrals to the experimental data, we have obtained exciton dispersion that possesses the same periodicity as the experimentally observed one. Also, our results provide an indirect confirmation that the 2D model is indeed a minimal one that describes available experimental data on exciton dispersion. Further, we have shown that exciton–exciton interactions produce negligible effects to the one-loop order. Due to that, we suggest that the influence of CT excitons in pentacene, which need to be taken into account, may be less important than indicated in previous studies. It would be interesting to see experimental data on exciton dispersion along $c^{*}$ axis and how this data would fit into the existing models. Also, it is important to understand how to improve the calculations based on BS equations to reach better agreement with experimental data on exciton dispersion and to test that approach for all four directions of the Brillouin zone considered in Reference [3]. Therefore, further experimental and theoretical work is necessary before drawing the final conclusion regarding the influence of CT excitons in pentacene. 

## References

[1] Forrest S.R. (2004). The path to ubiquitous and low-cost organic electronic appliances on plastic. Nature.

[2] Li G., Shrotriya V., Huang J., Yao Y., Moriarty T., Emery K., Yang Y. (2011). High-efficiency solution processable polymer photovoltaic cells by self-organization of polymer blends. Nat. Mater..

[3] Schuster R., Knupfer M., Berger H. (2007). Exciton Band Structure of Pentacene Molecular Solids: Breakdown of the Frenkel Exciton Model. Phys. Rev. Lett..

[4] Agranovich V., Ginzburg V. (1984). Crystal Optics with Spatial Dispersion and Excitons.

[5] Agranovich V. (2008). Excitations in Organic Solids.

[6] Rumyantsev V., Fedorov S., Gumennyk K., Sychanova M., Kavokin A. (2014). Exciton-like electromagnetic excitations in non-ideal microcavity supercrystals. Sci. Rep..

[7] Agranovich V., Bassani G. (2003). Thin Films and Nanostructures. Electronic Excitations in Organic Based Nanostructures.

[8] Schrter M., Ivanov S., Schulze J., Polyutov S., Yan Y., Pullerits T., Khn O. (2015). Excitonv–ibrational coupling in the dynamics and spectroscopy of Frenkel excitons in molecular aggregates. Phys. Rep..

[9] Tošić B., Pantić M., Lazarev S. (1997). Exciton concentrations in thin films. J. Phys. Chem. Solids.

[10] Roth F., Schuster R., Knig A., Knupfer M., Berger H. (2012). Momentum dependence of the excitons in pentacene. J. Chem. Phys..

[11] Roth F., Mahns B., Büchner B., Knupfer M. (2011). Exciton character in picene molecular solids. Phys. Rev. B.

[12] Cudazzo P., Gatti M., Rubio A. (2012). Excitons in molecular crystals from first-principles many-body perturbation theory: Picene versus pentacene. Phys. Rev. B.

[13] Cudazzo P., Gatti M., Rubio A., Sottile F. (2013). Frenkel versus charge-transfer exciton dispersion in molecular crystals. Phys. Rev. B.

[14] Cudazzo P., Sottile F., Rubio A., Gatti M. (2015). Exciton dispersion in molecular solids. J. Phys. Condens. Matter.

[15] Kronik L., Neaton J.B. (2016). Excited-State Properties of Molecular Solids from First Principles. Annu. Rev. Phys. Chem..

[16] Sharifzadeh S., Darancet P., Kronik L., Neaton J.B. (2013). Low-Energy Charge-Transfer Excitons in Organic Solids from First-Principles: The Case of Pentacene. J. Phys. Chem. Lett..

[17] Weinberg S. (2010). The Quantum Theory of Fields II.

[18] Wen X.G. (2007). Quantum Field Theory of Many Body Systems.

[19] Brauner T. (2010). Spontaneous Symmetry Breaking and Nambu–Goldstone Bosons in Quantum Many-Body Systems. Symmetry.

[20] Agranovich V., Toshich B. (1968). Collective Properties of Frenkel Excitons. Sov. Phys. JETP.

[21] Tyablikov S.V. (1967). Methods in the Quantum Theory of Magnetism.

[22] Frbrich P., Kuntz P. (2006). Many-body Green’s function theory of Heisenberg films. Phys. Rep..

[23] Auerbach A. (2012). Interacting Electrons and Quantum Magnetism.

[24] Manousakis E. (1991). The spin-1/2 Heisenberg antiferromagnet on a square lattice and its application to the cuprous oxides. Rev. Mod. Phys..

[25] Nolting W., Ramakanth A. (2009). Quantum Theory of Magnetism.

[26] Sandvik A.W., Kurkijärvi J. (1991). Quantum Monte Carlo simulation method for spin systems. Phys. Rev. B.

[27] Pantić M.R., Kapor D.V., Radošević S.M., Mali P.M. (2014). Phase diagram of spin-12 quantum Heisenberg J1–J2 antiferromagnet on the body-centered-cubic lattice in random phase approximation. Solid State Commun..

[28] Hofmann C.P. (1999). Spin-wave scattering in the effective Lagrangian perspective. Phys. Rev. B.

[29] Hofmann C.P. (2012). Low-temperature properties of two-dimensional ideal ferromagnets. Phys. Rev. B.

[30] Cornil J., Calbert J.P., Brdas J.L. (2001). Electronic Structure of the Pentacene Single Crystal: Relation to Transport Properties. J. Am. Chem. Soc..

[31] Stehr V., Pfister J., Fink R.F., Engels B., Deibel C. (2011). First-principles calculations of anisotropic charge-carrier mobilities in organic semiconductor crystals. Phys. Rev. B.

[32] Radošević S.M., Rutonjski M.S., Pantić M.R., Pavkov-Hrvojević M.V., Kapor D.V., Škrinjar M.G. (2011). The Neel temperature of a D-dimensional bcc Heisenberg antiferromagnet. Solid State Commun..

[33] Knupfer M., Berger H. (2006). Electronic Processes in Organic Solids. Chem. Phys..

[34] Marciniak H., Fiebig M., Huth M., Schiefer S., Nickel B., Selmaier F., Lochbrunner S. (2007). Ultrafast Exciton Relaxation in Microcrystalline Pentacene Films. Phys. Rev. Lett..

[35] Radošević S.M., Pantić M.R., Pavkov-Hrvojević M.V., Kapor D.V. (2013). Magnon Energy Renormalization and Low-Temperature Thermodynamics of O(3) Heisenberg Ferromagnets. Ann. Phys..

[36] Radošević S.M. (2015). Magnon-Magnon Interactions in O(3) Ferromagnets and Equations of Motion for Spin Operators. Ann. Phys..

[37] Leutwyler H. (1994). Nonrelativistic effective Lagrangians. Phys. Rev. D.

[38] Watanabe H., Murayama H. (2012). Unified Description of Nambu-Goldstone Bosons without Lorentz Invariance. Phys. Rev. Lett..

[39] Brauner T., Jakobsen M.F. (2018). Scattering amplitudes of massive Nambu-Goldstone bosons. Phys. Rev. D.

[40] Gongyo S., Kikuchi Y., Hyodo T., Kunihiro T. (2016). Effective field theory and the scattering process for magnons in ferromagnets, antiferromagnets, and ferrimagnets. Progr. Theor. Exp. Phys..

[41] Dyson F.J. (1956). General Theory of Spin-Wave Interactions. Phys. Rev..

[42] Dyson F.J. (1956). Thermodynamic Behavior of an Ideal Ferromagnet. Phys. Rev..

